# Ending the Pandemic: How Behavioural Science Can Help Optimize Global COVID-19 Vaccine Uptake

**DOI:** 10.3390/vaccines10010007

**Published:** 2021-12-22

**Authors:** Michael Vallis, Simon Bacon, Kim Corace, Keven Joyal-Desmarais, Sherri Sheinfeld Gorin, Stefania Paduano, Justin Presseau, Joshua Rash, Abebaw Mengistu Yohannes, Kim Lavoie

**Affiliations:** 1 Department of Family Medicine, Dalhousie University, Halifax, NS B3J 3T4, Canada; 2 Department of Health, Kinesiology and Applied Physiology, Concordia University, Montreal, QC H4B 1R6, Canada; simon.bacon@concordia.ca (S.B.); keven.joyal-desmarais@mail.concordia.ca (K.J.-D.); 3 Montreal Behavioural Medicine Centre, Montreal, QC H4J 1C5, Canada; lavoie.kim@uqam.ca; 4 Department of Psychiatry, University of Ottawa, Ottawa, ON K1Z 7K4, Canada; kim.corace@theroyal.ca; 5 Institute of Mental Health Research, University of Ottawa, Ottawa, ON K1Z 7K4, Canada; 6 Department of Family Medicine, University of Michigan, Ann Arbour, MI 48104, USA; ssgorin@med.umich.edu; 7 Department of Biomedical, Metabolic and Neural Sciences, University of Modena and Reggio Emilia, 41125 Modena, Italy; stefania.paduano@unimore.it; 8 Clinical Epidemiology, Ottawa Hospital Research Institute, Ottawa, ON K1Y 4E9, Canada; jpresseau@ohri.ca; 9 School of Epidemiology and Public Health, University of Ottawa, Ottawa, ON K1G 5Z3, Canada; 10 Department of Psychology, Memorial University of Newfoundland, St. John’s, NF A1B 3X9, Canada; jarash@mun.ca; 11 Department of Physical Therapy, Azusa Pacific University, Azusa, CA 91720, USA; ayohannes@apu.edu; 12 Department of Psychology, University of Quebec at Montreal (UQAM), Montreal, QC H2X 3P2, Canada

**Keywords:** COVID-19, vaccine acceptance, behavioural science, vaccine hesitancy, behaviour change

## Abstract

Governments, public health officials and pharmaceutical companies have all mobilized resources to address the COVID-19 pandemic. Lockdowns, social distancing, and personal protective behaviours have been helpful but have shut down economies and disrupted normal activities. Vaccinations protect populations from COVID-19 and allow a return to pre-pandemic ways of living. However, vaccine development, distribution and promotion have not been sufficient to ensure maximum vaccine uptake. Vaccination is an individual choice and requires acceptance of the need to be vaccinated in light of any risks. This paper presents a behavioural sciences framework to promote vaccine acceptance by addressing the complex and ever evolving landscape of COVID-19. Effective promotion of vaccine uptake requires understanding the context-specific barriers to acceptance. We present the AACTT framework (Action, Actor, Context, Target, Time) to identify the action needed to be taken, the person needed to act, the context for the action, as well as the target of the action within a timeframe. Once identified a model for identifying and overcoming barriers, called COM-B (Capability, Opportunity and Motivation lead to Behaviour), is presented. This analysis identifies issues associated with capability, opportunity and motivation to act. These frameworks can be used to facilitate action that is fluid and involves policy makers, organisational leaders as well as citizens and families.

## 1. Introduction

The COVID-19 pandemic has impacted nearly every human being in the world. There have been hundreds of millions of confirmed cases and millions of deaths, numbers which at the time of writing continue to increase [1]. COVID-19 has led to restricted freedom of movement, disrupted daily activities, impaired mental health, increased problematic substance use and reduced social interactions. For those who survive, COVID-19 has contributed to both short-term and long-term symptoms (“long covid”, [2]) such as weakness, shortness of breath, and exhaustion. The resultant increased workplace absenteeism and reduced productivity have had dramatic worldwide economic effects [3]. This pandemic has been a major and chronic life stressor; a threat to our way of life and our very survival.

Global, widespread uptake of COVID-19 vaccines is a major strategy for reducing hospitalizations, deaths and virus transmission and allowing a return to pre-pandemic ways of living. Due to the combined efforts of basic and clinical scientists, pharmaceutical companies and government agencies, we now have multiple COVID-19 vaccines that have been approved as both safe and effective for preventing severe disease, hospitalization and excess mortality [4]. Vaccine distribution systems have been implemented world-wide, although disparities in vaccine availability between low/middle income and high income countries have limited equitable distribution [5]. Even in high income countries, there are inequities in vaccine distribution by racial/ethnic, socioeconomic, age, and rural/urban differences [6]. However, the development of effective distribution systems is not enough. Even with widespread vaccine availability, the extent of uptake depends on vaccine acceptance at the individual and subgroup (e.g., social media group, political party allegiance) levels [7,8]. Vaccine acceptance has been identified by the World Health Organization (WHO) as a world-wide challenge [9]. Vaccine acceptance refers to people’s intention for, indecision about, or refusal of vaccination in response to an opportunity [10,11]. Vaccine acceptance begins with positive attitudes toward vaccination, leading to the intention to be vaccinated and ultimately ending in behavioural uptake. Views toward vaccination involve the balancing of beliefs that vaccination is needed and effective with concerns about the vaccine side-effects, safety, access, etc. [12]. Although many individuals (e.g., two-thirds in the US [13]) have accepted the vaccine, others may be unreached, or may experience ambivalence, a lack of concern, or active resistance [14]. Community immunity, through vaccination, which might require 55%-85% of the population to be vaccinated [15,16] with up-to-date doses, is ultimately an individual behavioral choice with a multitude of determinants that vary over time and across cultures, ethnicities, SES factors and political groupings [9,12,17,18,19,20,21].

Over the past two decades, behavioural scientists, those who study human behaviour, have been developing strategies to increase vaccine acceptance among parents of children and adolescents for mumps, measles, and rubella or human papillomavirus vaccines, as well as among adults for influenza, pneumonia and shingles vaccines [22,23,24,25]. This work has accumulated an extensive body of evidence about vaccine decision-making processes, and has helped to identify effective public health strategies to promote COVID-19 vaccination during this pandemic [12,26,27,28,29,30,31]. While we witness an increasing uptake of vaccinations as distribution has increased, we also observe continued variability in the percentage of vaccinated individuals both across and within countries. As well, the rates of acceptance of vaccination may be plateauing over time [32,33]. The goal of mitigating the negative health, personal and socio-economic consequences of COVID-19 might be unachievable unless vaccine acceptance can be optimized. A recent US Behavioural Science Task Force report categorized individuals as vaccine acceptors, the ‘moveable middle’ (akin to vaccine hesitant or ambivalent), and vaccine detractors [34]. They suggest vaccine acceptors be empowered as advocates, and strategies be put into place to minimize the negative impact of vaccine detractors. Regarding the ‘moveable middle’, strategies that reduce logistical and access barriers, leverage social influence and motivational frames, and build trust in vaccine safety, should be pursued.

Research into communication strategies to improve COVID-19 vaccine acceptance has also steadily been conducted throughout the pandemic. For example, large message-based studies in the US and in the UK have found that COVID-19 vaccination can be promoted by emphasizing how the vaccines impact people’s personal health outcomes, health outcomes for others around them, and, perhaps to a lesser extent, their countries’ economy [35,36,37,38]. Research has also examined how other factors, including the source of a message (e.g., medical expert, lay person [35]) and the emotions elicited (e.g., anger, guilt [38]) impact message effectiveness. This novel research is situated within a longer history of how communication-based techniques can be used to promote vaccine acceptance and reduce hesitancy [39,40].

Past research examining vaccination acceptance during the H1N1 epidemic provides a context for our current challenge. A systematic review of healthcare workers vaccination behaviour [41] validated the role of the Health Beliefs Model in predicting vaccination. Beliefs about seriousness, safety and effectiveness were associated with getting vaccinated. Interestingly, healthcare workers experienced barriers to vaccination including access, perceptions of a novel and rapidly developing vaccine, and impact of mass media. These latter issues have clear relevance to the current COVID-19 vaccination efforts. A similar systematic review of influenza vaccination uptake in healthcare providers also supported the use of behavioural frameworks [22] including the Health Beliefs Model and the Theory of Planned Behaviour.

Promoting behavior change to increase vaccine acceptance and uptake is complex and involves multiple, ecologic influences on individual decision making that require coordinated actions by individuals who function on multiple levels. These levels range from policymakers who make decisions for their respective jurisdictions (i.e., state/provincial, regional or country levels), to organisational leaders (e.g., company executives, managers, religious leaders, healthcare professionals) who may be important influencers among their employees/followers/ patients, and finally to citizens who, by accepting (or refusing) vaccination ultimately determine the success (or failure) of worldwide vaccination efforts. To our knowledge, no comprehensive framework has been proffered to coordinate and align both messages and activities across these levels. Such a framework is essential, as the interplay between individuals at the various levels is inherently complex, and complicated by the fact that information about vaccine efficacy and safety is emerging and evolving, resulting in the need for rapid adaptation (as evidenced by the recent emergence of the COVID-19 omicron variant). Further, vaccine supply chain challenges, such as distribution to low/middle income countries, export barriers imposed between countries, and distribution barriers within countries have been formidable [42]. As vaccines continue to become more available, additional challenges have arisen, such as determining optimal dosing schedules (required number of doses; time between doses), need for booster vaccinations, managing adverse events, and strategies for reopening economies. These multi-level challenges influence vaccine policies, implementation approaches at the organisational level, clinician advice, and vaccination behaviours; ultimately, these factors, if not managed appropriately, can result in confusion and suboptimal vaccination uptake.

The current suboptimal implementation of solutions at multiple levels (healthcare policy, community, organizations, families, and among individuals) is also limited when policy makers lack a clear understanding of the problem; i.e., understanding the specific reasons why certain individuals or groups hesitate or resist getting vaccinated. Common approaches such as incentives (lotteries, food) and education (instructing people to get vaccinated by espousing the benefits of vaccination), and communication strategies [35] may all play a role in optimization vaccination rates, but their individual success ultimately depends on the specific factors that are driving non-acceptance within different individuals or groups [43,44]. If people don’t care about vaccination, incentives may raise their (extrinsic) motivation to get vaccinated, but if people mistrust the government, incentives may be seen as coercive and reduce vaccination intention (by being perceived as infringing on individual rights). The issue is not so much about the solutions/strategies being ‘wrong’ or ‘insufficient’, but rather that different individuals and groups require different strategies. When interventions mismatch their target audience, they can be ineffective and even counterproductive [10,45]. Adapted, targeted and tailored approaches may be required to promote widespread vaccine acceptance [46,47].

## 2. Methods: Behavioural Science Frameworks for Optimizing Vaccine Acceptance

Combining knowledge on the determinants of vaccine acceptance with established frameworks for behaviour change, the behavioural sciences are well-positioned to support a coordinated approach to vaccine acceptance that can serve as a guide for multi-level stakeholders. Specifically, such frameworks could be helpful for identifying the different interventions or behaviours required to optimize vaccination uptake (ACTIONs), the individuals best positioned to deliver these interventions or engage in these behaviours (ACTORs), the recipients of these interventions or for whom the behaviours are performed (TARGETs), as well as the setting (CONTEXT) and timeline (TIME) within which actions should occur. The AACTT framework (Action, Actor, Context, Target, Time) [48], can help to organize and coordinate vaccination efforts across the multi-level stakeholders necessary for the success of the vaccine rollout. The AACTT framework can and should be used in a manner tailored to the realities of a given country or jurisdiction; approaches will need to be modified over time as new information on the success of implemented strategies emerges.

In Table 1, we illustrate how the AACTT framework could guide the behaviours of critical stakeholders involved in achieving optimal vaccination acceptance. We illustrate how policy makers, organisational leaders, and individuals/families can navigate specific challenges to vaccine acceptance. For policy makers, we outline how vaccines can be made available at the population level, with equitable distribution strategies. For organisational leaders, we describe how to reach marginalized populations by facilitating access. Among clinicians, we discuss how long-term trusted relationships with patients may be leveraged to provide supportive counselling to help those ambivalent explore concerns about, and promote the need for, vaccination. Finally, we outline the role that individuals and families can play in promoting vaccine acceptance. We suggest that the AACTT framework can be a guide to identify multi-level problems and specific solutions, including what needs to be done, by whom, for whom, when, and how.

Once we have defined the behaviour(s) to change using AACTT, understanding the processes by which behavior change occurs (in this case vaccine acceptance) is key. Recent work on the integration of various behaviour change theories and methods has led to a listing of the necessary ingredients to effectively promote behaviour change, resulting in the Capability-Opportunity-Motivation-Behaviour (COM-B) model ([49]; see Table 2). COM-B posits that behaviour change is influenced by three factors: (1) capability, having the physical and psychological abilities to engage in the behaviour; (2) opportunity, having the physical or social opportunities to engage in the behaviour; and (3) motivation, psychological processes that energise and direct behaviour such as the belief that a behaviour is important and/or socially desirable (see online Supplemental Table S1 for additional details on COM-B model). Identifying which COM-B domains are relevant for specific AACTTs can form the foundation for selecting fit-for-purpose tailored strategies to promote vaccine acceptance.

Case examples can be useful to understand how to implement the AACTT and COM-B frameworks in the context of optimizing vaccine acceptance. The COM-B could be used to inform whether there are capability, opportunity or motivation gaps that need addressing, and the AACTT framework can help identify the specific people, behaviours, time and place(s) of action. Consider the situation where, despite the availability of vaccines, there has been disproportionately low vaccine uptake in a particular neighborhood in a large urban city. In this city, vaccine clinics are located at all major hospital centers, are open from 8am to 6pm, 7 days a week, and vaccines are free of charge. Using the COM-B assessment at the individual level would first involve knowing the extent to which citizens in that neighborhood believe getting the vaccine is important and worthwhile. Surveys and/or interviews could inform understanding of the type of motivation (reflective or automatic) among community residents to be vaccinated. For instance, perceived fear of vaccination (e.g., side effects) might inform about automatic motivation. Reflective motivation might be evident in specific intention to be vaccinated, or in optimism that vaccination is the path to a return to normal. Noteworthy, reflective and automatic motivation might be inconsistent (e.g., fear side effects yet belief in the importance of vaccination), leading to ambivalence.

Second, we would need to determine the extent to which citizens are aware of the locations and hours of the vaccine clinics. Do they have the psychological capability to discern scientific advice from conspiracy theory and the physical ability to get to a vaccination clinic? Surveys could indicate whether they have the requisite information and access to transport to get to clinics.

Finally, we could determine the extent to which citizens can access transportation to attend a vaccination clinic, given their locations and opening hours (opportunity). Surveys could be administered to community members to assess work release policies, general hours of work, as well as the distance to healthcare clinics. In this example, through community surveys, imagine that, for the majority of the citizens living in this neighborhood, nonwork hours are primarily evenings and weekends, and that the closest major hospital centre is located about 30 min by car, 60 min by public transport. This suggests that the problem of low vaccine uptake among citizens in this neighborhood is likely attributed to a lack of opportunity to attend one of the vaccine clinics due to unavailable clinic hours and inconvenient access to a clinic. While motivated to get vaccinated and physically capable of receiving the vaccine, they have little opportunity to receive it.

With this information in hand, the AACTT framework could be leveraged to change vaccine distribution procedures as follows:

ACTION: change distribution procedures by implementing mobile vaccination clinics that can deliver vaccines evenings and weekends;

ACTOR: policy makers who could create mobile clinics for evenings/weekends or organizational leaders who could offer release time or establish vaccine clinics on site;

TARGET: citizens of the neighborhood who have been unable to access existing clinics;

CONTEXT: vaccine accessibility; bring the vaccines to neighborhood residents rather than expecting residents to attend hospital clinics;

TIME: Free mobile clinics in different sites around the community should be implemented immediately and be available evening and weekends.

The broad applicability of the COM-B and AACTT model to the decision-making and actions of all stakeholders (policy makers, organizational leaders and citizens) should be emphasized. For example, policy makers are responsible for coordinating access to and distributing vaccines at a population level. Their physical capability could reflect their ability to procure vaccines. Countries without the ability to produce vaccines locally must negotiate with manufacturers, compete with other countries that may have greater resources, or wait for assistance from others. For instance, Canada was slower than the US in rolling out first doses of vaccines due to the need for Canada to gain access to vaccines from other countries. Policy-makers’ psychological capability could reflect the extent to which they have the necessary advisors with the expertise to understand the rapidly-evolving science that is being used to inform policy. Opportunity factors may be enhanced or impeded at the community level. For example, the remoteness of certain geographical locations may make it difficult to get vaccines to people living in those regions. Difficulties with access to vaccines in remote locations may be overcome through support from urban communities (e.g., distribution of vaccines), rural communities (e.g., facilitating vaccine distribution throughout communities), and citizens (e.g., offering transportation to neighbors in need). Addressing Motivational factors for policy makers is complicated; automatic motivation may be influenced by implicit attitudes or emotions (e.g., fear of negative public reactions to implementing stricter policies). Further, reflective motivation involves balancing the need to protect public health with the practicalities of running a country, province or community. Harmonizing goals, messaging, expectations and approaches to facilitate vaccine acceptance or prevent the spread of COVID-19 may foster sustained reflective motivation.

COM-B has been useful to understand factors affecting vaccination uptake in marginalized populations. This work begins with qualitative research within a community to identify important barriers to vaccine uptake. In a study examining Black communities in Canada [50], anti-Black racism in health systems, concerns over representation and treatment of Black people in medical research, misinformation about vaccines, concerns about rapid development and issues related to accessibility were identified as barriers. These findings on the impact of mistrust were similar to those from recent longitudinal prospective population-based study of diverse Detroit, USA, residents [51]. While the perceived effectiveness and safety of the vaccine was associated with high acceptance, Black Detroiters were less accepting than other racial/ethnic subgroups. To address Capability factors, it would be important to strengthen culturally relevant knowledge and educational events that affirm cultural realities (Action, Target, Context). To address Opportunity factors, it would be important to address racism in the health systems, prioritize the inclusion of Black individuals in research, and prioritize rollout of vaccine within Black communities, as well as to administer vaccines in culturally sensitive ways such as allowing communities to determine when activities occur (Action, Target, Context, Time). Trust should be built by the engagement of Black community leaders (not government officials; Actor). Finally, the Motivation-related factors that could be addressed include acknowledging past mistreatment of Black communities and addressing fears of experiencing continued discrimination (Action, Actor, Target).

Among Indigenous communities in Canada [52], the impact of colonialism, structural racism and contemporary tensions between Indigenous nations and the government contribute to mistrust, skepticism and fear of vaccines promoted by the Canadian government. To address Capability factors, it would be important to strengthen culturally relevant knowledge and educational events that affirm pre-colonial realities (Action, Target, Context). To address Opportunity factors, it would be important to address racism in the health systems, access trustworthy sources of information such as Elders and Knowledge Keepers, and administer vaccines in culturally sensitive ways such as allowing communities to determine when and where activities occur (Action, Target, Context, Time). Ideally, these strategies could be endorsed by respected elders or community leaders (not government officials; Actor) to build trust. Finally, the Motivation-related factors that could be addressed include acknowledging past mistreatment of Indigenous populations and addressing fears of experiencing continued discrimination, and addressing broader health issues such as food insecurity and the contemporary issues concerning the residential school system (Action, Actor, Target).

The activities of organisational leaders can also be examined using the COM-B model. For example, leaders of community healthcare organizations, such as Federally Qualified Health Centers (FQHCs) in the US, that receive federal funds to offer primary care services to the poor and underserved, are often capable of accurately representing the needs of the groups that they serve [53]. They often know diverse populations’ needs and concerns very well, and what would be needed to address them (psychological capability through knowledge). Further, as leaders, they have access to physical resources within communities (and their organizations) to support vaccination efforts. By virtue of being ingrained in their communities, organisational leaders frequently encounter many opportunities, both physical and social, to increase uptake of vaccines within their constituencies in ways that policy makers do not (e.g., having conversations with community members and being presented with events in the communities through which vaccination can be promoted, and advocating with other community leaders for more vaccination resources). Regarding motivational factors, like policy makers, organizational leaders have their own specific belief systems/ideologies that guide their decision-making and behaviours, such as trust in governmental institutions. They are also guided by the ‘agendas’ of the individuals around them, including their staff and their boards of directors. These personal (or group-level) motivations could either undermine or encourage their actions regarding community vaccination efforts. Uncovering these motivators is important to developing an action plan to overcome barriers to increasing vaccination behaviors in their communities.

Finally, COM-B has relevance to increasing vaccine acceptance directly among citizens and families in general. Physical capability can be hindered among those who live with disabilities or have mobility issues, and psychological and cognitive belief systems can be major barriers to vaccination, particularly in the context of rampant misinformation. Opportunity factors are also important, especially for gaining physical access to vaccination centres as seen in our example above. Additionally, individuals tend to gravitate toward social groups that share the same opinion, and these individuals can either encourage or discourage vaccine uptake (social opportunity). Finding ways to strengthen the social influence of the scientific community without threatening existing social norms is critical, as is leveraging members of social networks who have been vaccinated (e.g., sharing positive experiences). Most important at the individual level could be motivation. Those with pro-vaccine attitudes and beliefs will accept to be vaccinated, or actively pursue opportunities to be vaccinated. As more and more people become vaccinated, the unconcerned, hesitant, and the resistant will become less common [14]. However, over time, the smaller number of unvaccinated groups and individuals may become stigmatized, however, as smokers have become, potentially reducing the motivation to vaccinate [54]. Finding ways of strengthening the motivation to get vaccinated while continuing to monitor the effectiveness of policies and programs to encourage vaccination remains a constant challenge for policy makers and leaders. This challenge is especially great if high rates of vaccination are needed for countries/regions to relax restrictions and reopen their economies and borders. Using the COM-B framework can be a useful guide: targeting automatic motivation can involve using public health messages that avoid forceful/coercive language known to elicit negative feelings and resistance [55]. Reflective motivation can also involve strengthening intentions (and helping people act on their intentions by making plans) and capitalizing on beliefs related to positive intentions.

## 3. Discussion

Now that biological science has created vaccines against COVID-19, pharmaceutical companies/researchers have produced and tested vaccines for efficacy and safety, and epidemiologist/public health officers are tracking Rt rates, cases, hospitalization and death, it is now a matter of getting needles into arms. Policy makers face issues of access and distribution. However, addressing these alone will not be enough to achieve optimal uptake of the vaccine. Deciding to get vaccinated (or not) remains an individual choice in most jurisdictions. As such, behavioural science can play a significant role in informing how best to address vaccine hesitancy and increase vaccine confidence as a means to ending the pandemic.

We have described two established behavioural frameworks that we believe can facilitate the efforts of policy makers and organisational leaders to encourage individuals to overcome vaccine hesitancy and support their decision to be vaccinated. These frameworks can inform the identification and implementation of strategies to overcome the known and future barriers to vaccination). The AACTT framework identifies the goal to be achieved (Action), the individuals who need to be involved in the intervention (Actors), the ecologic Context, as well as the Target and Timing of an intervention. The COM-B model can further aid in the identification of factors associated with capability, opportunity and motivation.

Application of the AACTT and COM-B frameworks does not ensure the success of chosen interventions, particularly in the absence of optimal implementation. It is important to monitor the process through which the chosen intervention is delivered, and outcomes achieved.

## 4. Future Directions

Prospectively designed population-based studies, combined with local clinical data are required to determine the efficacy of these two behavioral frameworks, the AACTT and the COM-B. The systematic assessment of both population-and local-level data within the context of these two behavioral frameworks will inform evidence-based care to those with vaccine hesitancy, and determine the uptake rate of COVID-19 vaccines. The collection of such data in real-time will ultimately generate practice-based evidence that can improve public health outcomes [56].

We encourage the joint use of these two well-established behavioral frameworks, the AACTT and the COM-B model. Together, these frameworks can help maximize vaccination rates by capitalizing on decades of behavioural science research.

## Figures and Tables

**Table 1:** The AACTT Framework Applied to Vaccine Uptake.

	Policy Makers	Organisational Leaders	Individual and Family
	Example: Increase Rapid Vaccine Supply across the Population	Example: Increase Access for Marginalized Populations	Example: Get Vaccinated and Encourage Others
**Action** **(what needs to be done)**	- Approval and funding of vaccines including subsidies for those unable to pay. - Promotion of policies authorizing vaccine distribution. - Provision of multiple vaccine sites, promotion of pop-up centers and distribution through established healthcare sites (e.g., departments of public health, physicians’ offices, Federally Qualified Health Centers FQHC’s). - Vaccine availability a at community centres, places of worship (e.g., mosque, synagogue, church). - Work with community/religious leaders to promote vaccination; provide incentives that are in line with the values of the community. - Present health policy recommendations to the public. - Enforcement of public health orders.	- Organize distribution of vaccines at community centres and/or places of worship (e.g., mosque, synagogue, church). - Work with other community/religious leaders to promote vaccination; provide incentives that are in line with the values of the community. - Advocate for additional vaccine sites, for monetary coverage of the cost of vaccines for the uninsured. - Assign healthcare providers from that community to deliver vaccines. - Disseminate vaccine information. - Offer work release time to obtain a vaccine. Offer incentives for vaccination. Align internal policies with increasing vaccination by employees.	-Attend a vaccination appointment as opportunities arise. - Promote vaccination amongst personal social network. - Talk favorably about vaccine scheduling and dosing among family and friends. - Promote objective information and be mindful to dispel sensationalist depictions.
**Actor** **(who needs to do it)**	- Political leaders. - Public health officials - Policy makers.	- Community/religious and industry leaders (e.g., imam, priest, rabbi). - Community members who are healthcare providers.	- Each individual including partners, family members and social network members, as well as parents.
**Context** **(where it needs to be done)**	- Decision making context such as legislature or policy group.	- Hospitals, community health clinics (e.g., FQHCs), worksites. - Community centres/events. - Places of worship.	- Ongoing daily life as information about vaccination is available. - Information campaigns and public health updates.
**Target** **(the intended recipient of the actions)**	- Citizens matching the current rollout plan (eg., by age or by risk)	- Employees. - Those who live in the community who meet the current eligibility criteria (e.g., by age or by risk).	- The individual citizen, their friends, family, community and dependents.
**Time** **(when the actions should occur)**	- Establish specific times for follow up processes, being sensitive to “hotspots”. - Set time to re-evaluate the policy as implementation proceeds, and data emerge regarding success (e.g., case counts). - Establish timing for routine monitoring of the population vaccine registry to establish need for changes (e.g., weekly, monthly, depending on the overall case counts and the rates in the “hotspots.”).	- Establish timetable for routine collection and monitoring of data on the impact of internal policies, and combined community efforts. - Establish timetable to monitor the local vaccine registry for changes (e.g., in the electronic medical record or in employee records). - Determine when to conduct regular evaluations of implementation success on vaccine uptake and cost.	- When new vaccines distribution sites are offered (age-based rollout; pop up vaccination centres, etc).

**Table 2:** The Capability, Opportunity and Motivation (COM-B) Model.

Capability	Policy Makers	Organisational Leaders	Individual Citizens
**Physical Capability**	Ability to physically perform a behaviour (e.g., biological factors, physical ability)		
**Psychological Capability**	Having the knowledge (e.g., accurate information) and mental functioning (e.g., cognitive skills) to enact a behaviour		
**Opportunity**			
**Physical Opportunity**	Having the resources (e.g., time, money) and a physical environment (e.g., access to transportation) that enable the behaviour		
**Social Opportunity**	The presence of social and cultural norms (e.g., supportive others) that support the behaviour		
**Motivation**			
**Automatic Motivation**	Psychological factors that influence behavior outside of deliberate thought (e.g., emotions, habits, instinct, reinforcement)		
**Reflective Motivation**	Psychological factors that stem from people’s explicit reasoning/thinking (e.g., setting intentions/goals, social and professional role, self-efficacy, beliefs about consequences of enacting the behaviour)		

## Data Availability

There are no data in this work.

## Supplementary Materials

The following are available online at https://www.mdpi.com/article/10.3390/vaccines10010007/s1, Table S1: Application of behaviour change theory and the behaviour change technique taxonomy to overcoming barriers and encouraging vaccination uptake.
