# Supplementary material for: Ending the Pandemic: How Behavioural Science Can Help Optimize Global COVID-19 Vaccine Uptake
Source: Vaccines (Basel). 2021 Dec 22;10(1):7. doi: 10.3390/vaccines10010007 (PMC8777721; doi:10.3390/vaccines10010007)
Supplement: Supplementary file 1 [file vaccines-10-00007-s001.zip › vaccines-1484408-supplementary.pdf]

**Online supplement S1**

Supplemental Table S1 was constructed as an illustrative example of the ways in which behaviour change theory can be used to facilitate Covid-19 vaccination uptake. Impediments to Covid-19 vaccination uptake most commonly endorsed within the literature have been detailed in accordance with the Capability-Opportunity-Motivation Behaviour (COM-B) model of behaviour change.[1,2] Pertinent intervention functions to address deficits in capabilities, opportunities and motivation are depicted in accordance with the Behaviour Change Wheel.[3] The likely mechanism of action underlying the intervention function was selected based on the taxonomy presented by Carey et al.[4] The most applicable behaviour change techniques likely to induce the mechanism of action was drawn from the behaviour change technique taxonomy,[5] and linked to mechanisms of action in accordance with Johnson et al. (2020).[6] Supplemental Table S1 was designed to serve as an initial attempt to depict the application of behaviour change theory to vaccination uptake at the level of the individual citizen. We acknowledge that components of the COM-B model interact to influence vaccination uptake and the optimal approach will be based on techniques that are skilfully tailored to the individual citizen. We also acknowledge that considerable heterogeneity exists within vaccine hesitancy that may influence the optimal application of behaviour change techniques. For example, Fournet et al.[7] proposed a model that identified four under-protected vaccination groups, including 1) the hesitant who are concerned about safety issues and unsure about needs, procedures and timing of vaccines; 2) the unconcerned who consider immunization a low priority and perceive low risk of vaccine-preventable diseases; 3) the poorly reached who have limited or difficult access to service; and 4) the active resisters for whom personal, cultural, or religious beliefs discourage vaccination. Identification of such groups can assist with the selection of interventions to improve vaccine uptake. Finally, we recognize that the timing and method of delivering behaviour change techniques is of

crucial importance. Structured approaches to address vaccination concerns have been developed with variable results (refer to Brewer et al.[8]). It is clear that decision-making around vaccination is a complex process, and uptake can be improved through the application of behaviour change techniques in a manner that activates mechanisms of action that are consistent with behaviour change theory.

Supplemental Table S1.

*Application of behaviour change theory and the behaviour change technique taxonomy to overcoming barriers and encouraging vaccination uptake*

**Target Behaviour:** Facilitate attendance at one or two vaccination appointments to receive the vaccine

| COM-B components | What needs to happen for vaccination to occur? | Is there a need for change? | Intervention functions [corresponding behaviour change theory] | Mechanisms of Action to address change | Behaviour change techniques |
|---|---|---|---|---|---|
| ***Physical capability*** | Have the physical skills to receive a vaccination | Most of the population have the physical capability to receive a vaccination; however, some do not (e.g., those faced with a decision to come off critical immunosuppressants to receive a vaccine). | | | |
| ***Psychological capability*** | Knowledge of when and how to schedule and obtain a vaccination. | Vaccine knowledge is variable, particularly given misinformation,[9-11] and is associated with intention to vaccinate.[12] Individuals may require change in their knowledge. | Education about local processes. | 1. Knowledge.<br><br>2. Behavioural cueing. | 1. Instruction on how to schedule and receive the vaccination.<br>2. Effective system for scheduling prompts and reminders for vaccination. |
| | Comprehend Covid-19 as a serious potential threat to self or others. | Perceived severity of Covid-19 is associated with intention to vaccinate.[12] Not all individuals believe that Covid-19 is a serious disease. | Education about the risk and severity of Covid. [Health Belief Model] | 1. Beliefs about consequences. | 1. Information about health and emotional consequences associated with Covid.<br>1. Salience of consequences. |
| | Understand the importance of vaccine as a method to provide controlled and standardized protection. | Efficacy of the Covid-19 vaccine is a strong predictor of intention to vaccinate,[13,14] but not all individuals believe that obtaining a vaccine is required to bring about meaningful change. | Education about the benefits of the vaccine. [Health Belief Model] | 1. Feedback processes<br><br>2. Behavioural regulation. | 1. Provide accurate feedback on outcomes of behaviour in terms of efficacy (e.g., reducing risk of contraction and transmission) and duration of immunity.<br>2. If possible, develop discrepancy between current behaviour and goal of reducing transmission of Covid. |
| | Perceived confidence to obtain vaccine. | Vaccine self-efficacy is associated with vaccine intentions,[15] yet many lack confidence in their ability to schedule and obtain the vaccine. | Training, modelling, and enabling. [Social Cognitive Theory]. | 1. Behavioural regulation.<br><br>2. Beliefs about capabilities.<br><br>3. Skills. | 1. Action planning.<br>1. Problem solving (e.g., scheduling, transit, and cost).<br>2. Focus on past successes.<br>2. Behavioural practice or exposure (in-vivo or imaginal).<br>3. Problem solving. |
| ***Physical opportunity*** | Have access to an available vaccine | Vaccines are not equitably available for all | Enablement by increasing accessibility and opportunity. | 1. Behavioural cueing. | 1. Restructuring the physical environment, bringing vaccine to those in need, and |

| | | | | | |
|---|---|---|---|---|---|
| | | demographics,[16] and this needs to change. | [Behaviourism] | 2. Environmental context and resources | incorporating place-based vaccine prioritization.<br>2. Problem solving to overcome barriers. |
| | Have peer or social support needed to obtain the vaccine. | Not everyone has the social support necessary to assist in facilitating vaccination. | Enablement. | 1. Social influences. | 1. Encourage use of social support network to receive tangible and emotional supports. |
| ***Social opportunity*** | See respected others receiving vaccine (e.g., health officials, friends, family members, celebrities). | Endorsement by credible sources increases vaccine intention,[14,17] yet widespread modelling has not been optimised. | Modelling<br>[Social norm theory] | 1. Social learning / imitation<br>2. Attitudes towards vaccination | 1. Demonstration of the behaviour, particularly with exemplars that resonate with the citizen.<br>2. Use of credible sources. |
| | Have a normative social belief that obtaining the vaccine is "the right thing to do." | Widespread normative social beliefs about the vaccine have not taken been optimised. | Education and modelling<br>[Social norm theory] | 1. Intention<br>2. Social norms | 1. Information about others approval.<br>2. Facilitate favourable social comparisons. |
| ***Reflective motivation*** | Beliefs that benefits associated with vaccination outweigh potential side effects. | Viewing benefits of vaccination as outweighing costs is associated with vaccine acceptance,[13,14] yet messaging about vaccines could make adverse effects appear more salient and outweigh potential benefits creating an availability bias.[18] | Education and persuasion. | 1. Motivation<br>2. Attitudes towards vaccination.<br>3. Beliefs about consequences. | 1. Weigh pros and cons of vaccination.<br>2. Positive reframing of mild adverse effects.<br>3. Comparative imaging of vaccine side effects and contracting or transmitting of serious Covid-19 symptoms.<br>3. Reframing by highlighting the number of people who do not experience serious adverse effects. |
| | Little to no resistance or hesitancy towards receiving the vaccine. | Vaccine hesitancy is associated with conspiratorial and religious beliefs, and mistrust of authoritative members of society.[12,19] Such hesitancy needs to change. | Education, persuasion, and incentivisation. | 1. Explore values that can be tied to vaccination.<br>2. Self-image<br>3. Beliefs about vaccine development and risk. | 1. Facilitate a perception of one's identity that is associated with being vaccinated.<br>2. Explore ambivalence to reduce psychological reactance and focus on reframing of beliefs.<br>3. Explain speed of vaccine development and approval.<br>4. Correcting misinformation |
| ***Automatic motivation*** | Does not experience overwhelming feelings of fear, anxiety, or worry towards receiving the vaccine. | Some individuals experience overwhelming fear or anxiety towards receiving the vaccine or needles. | Education and training. | 1. Knowledge.<br>2. Behavioural regulation. | 1. Address common misconceptions about vaccine adverse events.<br>2. Prepare for exposure to feared stimuli given that corrective information can cause further reactance among anxious individuals. |
| **Behavioural diagnosis of the relevant COM-B components:** | **A custom approach to be tailored for the specific person, sub-group of interest based on the barriers and enablers that they are reporting.** | | | | |

## References


1. Michie S, Atkins L, West R. The behaviour change wheel: A guide to designing interventions. Great Britain: Silverback Publishing; 2014.
2. West R, Michie S. A brief introduction to the COM-B Model of behaviour and the PRIME Theory of motivation. Qeios 2020.
3. Michie S, van Stralen MM, West R. The behaviour change wheel: A new method for characterising and designing behaviour change interventions. Implementation science : IS 2011;6:Artcile 42.
4. Carey RN, Connell LE, Johnston M, et al. Behavior Change Techniques and Their Mechanisms of Action: A Synthesis of Links Described in Published Intervention Literature. Annals of behavioral medicine : a publication of the Society of Behavioral Medicine 2019;53:693-707.
5. Michie S, Richardson M, Johnston M, et al. The behavior change technique taxonomy (v1) of 93 hierarchically clustered techniques: building an international consensus for the reporting of behavior change interventions. Annals of behavioral medicine : a publication of the Society of Behavioral Medicine 2013;46:81-95.
6. Johnston M, Carey RN, Connell Bohlen LE, et al. Development of an online tool for linking behavior change techniques and mechanisms of action based on triangulation of findings from literature synthesis and expert consensus. Translational behavioral medicine 2021;11:1049-65.
7. Fournet N, Mollema L, Ruijs WL, et al. Under-vaccinated groups in Europe and their beliefs, attitudes and reasons for non-vaccination; two systematic reviews. BMC public health 2018;18:Article 196.
8. Brewer NT, Chapman GB, Rothman AJ, Leask J, Kempe A. Increasing Vaccination: Putting Psychological Science Into Action. Psychological science in the public interest : a journal of the American Psychological Society 2017;18:149-207.
9. Hotez P, Batista C, Ergonul O, et al. Correcting COVID-19 vaccine misinformation: Lancet Commission on COVID-19 Vaccines and Therapeutics Task Force Members. EClinicalMedicine 2021;33:100780.
10. Loomba S, de Figueiredo A, Piatek SJ, de Graaf K, Larson HJ. Measuring the impact of COVID-19 vaccine misinformation on vaccination intent in the UK and USA. Nature human behaviour 2021;5:337-48.
11. Wilson SL, Wiysonge C. Social media and vaccine hesitancy. BMJ global health 2020;5.
12. Ruiz JB, Bell RA. Predictors of intention to vaccinate against COVID-19: Results of a nationwide survey. Vaccine 2021;39:1080-6.
13. Kaplan RM, Milstein A. Influence of a COVID-19 vaccine's effectiveness and safety profile on vaccination acceptance. Proceedings of the National Academy of Sciences of the United States of America 2021;118.
14. Kreps S, Prasad S, Brownstein JS, et al. Factors Associated With US Adults' Likelihood of Accepting COVID-19 Vaccination. JAMA network open 2020;3:e2025594.
15. Guidry JPD, Laestadius LI, Vraga EK, et al. Willingness to get the COVID-19 vaccine with and without emergency use authorization. American journal of infection control 2021;49:137-42.
16. Bibbins-Domingo K, Petersen M, Havlir D. Taking vaccine to where the virus is—equity and effectiveness in coronavirus vaccinations. JAMA Health Forum; 2021: American Medical Association. p. e210213.
17. Daly M, Jones A, Robinson E. Public Trust and Willingness to Vaccinate Against COVID-19 in the US From October 14, 2020, to March 29, 2021. Jama 2021;325:2397-9.

18. Rief W. Fear of Adverse Effects and COVID-19 Vaccine Hesitancy: Recommendations of the Treatment Expectation Expert Group. JAMA Health Forum 2021;2:e210804.
19. Murphy J, Vallières F, Bentall RP, et al. Psychological characteristics associated with COVID-19 vaccine hesitancy and resistance in Ireland and the United Kingdom. Nature communications 2021;12:29.
